# Template CoMFA Generates Single 3D-QSAR Models that, for Twelve of Twelve Biological Targets, Predict All ChEMBL-Tabulated Affinities

**DOI:** 10.1371/journal.pone.0129307

**Published:** 2015-06-12

**Authors:** Richard D. Cramer

**Affiliations:** Discovery Division, Certara Corporation, Santa Fe, New Mexico, United States of America; University of Bologna & Italian Institute of Technology, ITALY

## Abstract

The possible applicability of the new template CoMFA methodology to the prediction of unknown biological affinities was explored. For twelve selected targets, all ChEMBL binding affinities were used as training and/or prediction sets, making these 3D-QSAR models the most structurally diverse and among the largest ever. For six of the targets, X-ray crystallographic structures provided the aligned templates required as input (BACE, cdk1, chk2, carbonic anhydrase-II, factor Xa, PTP1B). For all targets including the other six (hERG, cyp3A4 binding, endocrine receptor, COX2, D2, and GABAa), six modeling protocols applied to only three familiar ligands provided six alternate sets of aligned templates. The statistical qualities of the six or seven models thus resulting for each individual target were remarkably similar. Also, perhaps unexpectedly, the standard deviations of the errors of cross-validation predictions accompanying model derivations were indistinguishable from the standard deviations of the errors of truly prospective predictions. These standard deviations of prediction ranged from 0.70 to 1.14 log units and averaged 0.89 (8x in concentration units) over the twelve targets, representing an average reduction of almost 50% in uncertainty, compared to the null hypothesis of “predicting” an unknown affinity to be the average of known affinities. These errors of prediction are similar to those from Tanimoto coefficients of fragment occurrence frequencies, the predominant approach to side effect prediction, which template CoMFA can augment by identifying additional active structural classes, by improving Tanimoto-only predictions, by yielding quantitative predictions of potency, and by providing interpretable guidance for avoiding or enhancing any specific target response.

## Introduction

An improved method for predicting the interactions of any small organic molecule (ligand) with any possible biological target should provide great value in the discovery, regulation, and practical application of new substances, including but not limited to pharmaceuticals [1, 2]. Two general approaches can be distinguished, physical and statistical, each with strengths and limitations. The physical approach of directly simulating molecular interactions promises theoretical certainty and general applicability, but in practice evidently struggles within the immense physical and biological state space that these simulations should explore[3], for its predictions are too computationally demanding for extensive application and in quality often not distinguishable from random [4]. The statistical strategy seeks correlations between observed biological interactions and other ligand descriptors [5], most often as similarities derived from the occurrence frequencies of structural fragments [6]. These relationships are easily obtained and often usefully accurate[1, 2, 7], but do not directly invoke causation and so have uncertain structural and biological scopes of applicability, and provide little structural guidance.

Therefore approaches that might blend the strengths and blunt the limitations of these two strategies are attractive. One such approach is 3D-QSAR [8, 9], which focuses statistical correlations on those ligand physical properties that can be causatively related to biological interactions. More exactly, with most biological interactions being non-covalent, it can only be differences among ligands’ non-covalent fields that cause the observed differences in their biological effects. In practice, such field differences are usually expressed as the intensities of electrostatic and van der Waals potential fields exerted by each ligand at the intersections of a fixed Cartesian lattice. Partial least squares (PLS) then yields a statistical model, whose coefficients, being defined spatially, can be contoured to provide an informative visual representation. Applications of 3D-QSAR (in particular CoMFA [10]) to molecular discovery are the subjects of many thousands of publications.

However 3D-QSAR has been challenging to practice. Its results critically depend on “alignment”, which includes how each ligand of interest is positioned within the lattice along with the conformational uncertainty of most ligands. When possible, ligands are usually superimposed in their target-bound geometries, as obtained from crystallography or inferred from docking calculations. Or, if no target structure is available, “ligand-based” alignment approaches can be tried, either by overlaying characteristic molecular substructures, or by identifying a common geometry among such important molecular features as hydrogen-bonding atoms and ring centroids. However the relative disposition of ligand side chains remains undetermined and the combined modeling of multiple diverse ligand series is especially problematic. Whatever the alignment approach, its results are often inspected individually, and perhaps further adjusted manually, in hopes of obtaining a statistically more impressive model providing more accurate predictions of biological affinity. All this pre- and post-processing is tedious and potentially subjective, limiting the sizes and scopes of the datasets to which 3D-QSAR has usually been applied.

A search for refinements that would overcome these alignment challenges has long been the author’s major activity [11, 12], with template CoMFA as its latest outcome [13]. Template CoMFA has just two inputs, one or more “aligned templates”, structures whose relative 3D geometries have somehow already been defined, and a training set of biodata observations, biological activities of structures described only by their “2D” connectivities. Template CoMFA generates a 3D alignment for any “candidate” structure, either one within a training set or one whose activity is to be predicted, starting with its Concord structure, by comparing its connectivity with that of each of the input templates, starting from every pairing of suitable “anchor bonds”, and seeking a “best match” of an anchor-bond connected atom chain within this candidate to an anchor-bond connected atom chain within one of the templates. Then, the anchor bond selected within the candidate is superimposed onto the anchor bond selected within the selected template, and the relative coordinates of the “matching” atoms from the “best matching” template are copied to their corresponding atoms within the candidate. Finally the non-corresponding atoms remaining within the candidate are positioned using a canonical “topomer” protocol[14].

Template CoMFA alignment is very fast and by default entirely automatic and objective. In essence its aligned template inputs embody user-specified rules, because their uniform application first to training set structures and then to the structures being predicted is rigidly enforced. While template CoMFA was developed with structurally local models and lead optimization in mind, these advantageous properties suggested its possible further application to the much larger and more structurally diverse data sets involved in off-target prediction. Initial trials confirmed: that, with crystallographic templates, biodata from multiple diverse structural series could be pooled to yield a single 3D-QSAR model for a target of interest [13]; that this useful result was obtained for all of 114 biological targets tried [15]; and that template CoMFA models of three targets were of comparable statistical quality and arguably superior interpretability to models whose training set alignments were defined by X-ray crystallography [16].

Here are reported further investigations of template CoMFA’s broader applicability, addressing such specific concerns as:

- Can template CoMFA models be obtained from training sets whose structures mostly lack any obvious homologies whatsoever?
- Can stable and robust template CoMFA models be obtained from input templates whose alignment is purely ligand-based, and therefore far more uncertain and subjective, than X-ray-aligned input templates?
- How dependent are template CoMFA models and their predictions on the number or variety of their input templates?
- How dependent are the models and their predictions on the sizes of their training sets?
- How accurate are template CoMFA predictions? How do its predictions compare with those from the established approach based on substructure frequencies and counts, particularly for the binary active/inactive decisions that characterize off-target prediction or virtual screening?

Positive findings from these investigations suggest that template CoMFA may constitute a useful advance in predicting the occurrence and strength of interactions between arbitrary ligands and arbitrary targets.

## Materials and Methods

To evaluate an off-target prediction methodology that specifically considers affinity as well as occurrence, large and diverse training sets that include curated affinity measurements are also necessary. The exemplary ChEMBL data base was used herein [17]. Yet the inherent limitations of such literature compilations also need mention. Their sources are strongly biased toward highly active ligands, even though most structures actually have very little affinity for most targets. And variability among laboratories in the design and execution of nominally equivalent assays can produce substantial discrepancies in the affinities reported for the same structure.

**Fig 1:**
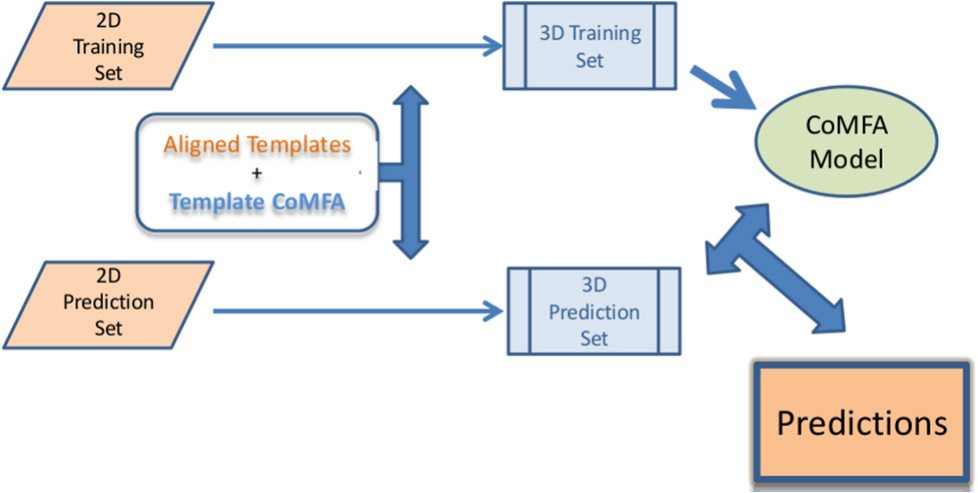
The Template CoMFA Workflow.

A high-level schematic of the template CoMFA workflow used in all of this work appears in Fig 1. As shown there, a template CoMFA program uses the 3D information provided in a set of aligned templates provided by the user to convert sets of 2D structures into 3D structures. The 3D structures designated for training are used to construct a CoMFA model, and finally predictions are obtained by applying that CoMFA model to the template CoMFA aligned 2D structures. The details of its individual processes can be found in previous publications [13, 12, 14]. Evidently, and as already emphasized, by design this workflow is inflexible; thus results for a given training set can be affected only by varying the initial choices of templates. Therefore, these studies varied primarily in which of seven protocols was used to generate templates. For convenient reference, these protocols are labeled A through G. Also the “standard” protocol B was applied to two other partitionings of the ChEMBL data into training and prediction sets. The rationale for each of these protocols is presented in the Results and Discussion sections.

The twelve targets, listed in Table 1, were chosen while considering functional and structural diversity, current interest, and the availability of ample biodata. For each target, all of its IC50 and Ki “Target Associated Biodata” were downloaded from ChEMBL. Because of the above-mentioned variation in the affinity values (CHEMBL_VALUEs) that different assay procedures (ASSAY_CHEMBLIDs) often reported for the same compound, all such duplicate measurements were tabulated by pairs, sorted by reported affinity difference, and inspected. Any biodata observation, anywhere in a biodata table, whose ASSAY_TYPE was repeatedly associated with affinity differences greater than 2.0 in this sorted table was dropped. (Note, for example, that without this filtration to remove all values from discordant sources/assays, no model whatsoever could be obtained for carbonic anhydrase II.) Counts of biodata observations downloaded, and then after removal of observations either lacking a CHEMBL_VALUE or filtered out as described appear in the second and third columns of Table 1.

**Table 1:** Properties of the biological data sets.

Target Name	# .pdb tmpls	# ChBL vals	# ChBL vals used^a^	# skels^b^	SD, ChBL vals used	SD, TC pred, prtcl B	SD, LOO Xval, prtcl B	s, MW MR	SD, NN pred	r^2^, TC pred vs NN pred	ChEMBL Tgt ID
**bace**	13	4590	4070	519	1.23	0.87	0.87	1.21	0.88	0.289	4822
**cdk2**	20	2234	545	185	1.05	0.90	0.87	1.03	0.84	0.187	301
**chk1**	43	4340	1932	452	1.30	0.97	1.01	1.20	1.02	0.224	4630
**CA-II**	65	8623	5981	127	1.12	0.83	0.83	1.05	0.84	0.129	206
**COX2**	0	8919	3744	297	1.20	0.98	0.97	1.19	1.01	0.248	230
**CYP3A4**	0	7198	3224	957	0.94	0.79	0.79	0.91	0.85	0.313	340
**D2A**	0	8860	5750	307	1.09	0.82	0.87	1.06	0.92	0.214	217
**ER**	0	3246	1869	434	1.43	0.94	0.98	1.32	0.94	0.244	4296
**facXa**	12	6273	5471	1012	1.58	1.14	1.13	1.54	1.04	0.231	244
**GABAa**	0	3449	1691	7	1.33	0.99	1.00	1.28	0.97	0.256	1907607
**hERG**	0	7690	4871	1268	0.95	0.82	0.82	0.95	0.80	0.208	240
**ptp1b**	36	4682	2709	577	0.99	0.67	0.70	0.88	0.62	0.179	335

See text for details.

^a^See Materials and Methods section for discussion

^b^Such a "reduced skeleton" is obtained for a structure by (1) removing hydrogens; (2) converting all remaining atom and bond types to "Any"; (3) iteratively deleting terminal atoms until no more exist (leaving only the connected rings)

Each of these twelve sets was divided evenly into training and prediction sets, in a uniform yet random fashion, simply by putting the odd-numbered structures into the training set and the even-numbered into the prediction set.

**Fig 2:**
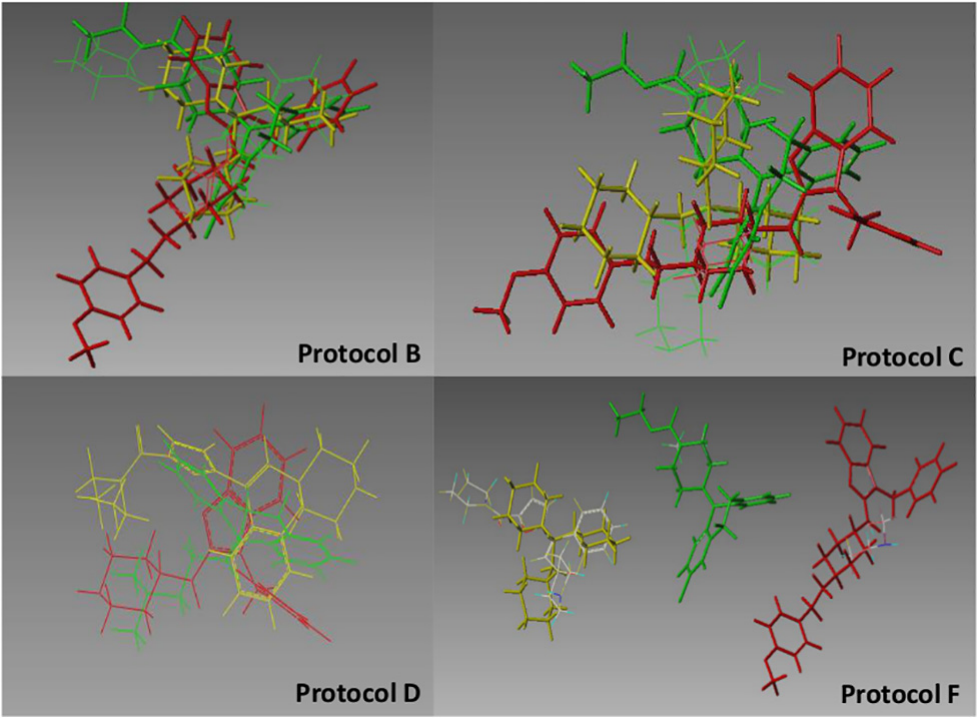
Input template alignment alternatives for the hERG target. The three structures in each image are loratadine (green), perhexilene (yellow), and astemizole (red). Each one-letter label refers to the protocol that generated the alignment. The protocol F image also includes the best-matching 3D structure, among the training set, which the template CoMFA program generated for each of these three templates.

Templates for these targets had two sources. One was all of the .pdb entries for the target that were referenced by the www.bindingdb.org URL [18], using a procedure described elsewhere [13] to overlay the .pdb structures and extract a template ligand from each. Whenever available for a target (as shown in the first column in Table 1), these templates enabled protocol A. For the other protocols, the template source was three known ligands, chosen for structural diversity and/or therapeutic prominence, from among the .pdb structures when available, or else from literature sources, Wikipedia in particular. The same triplet of ligand structures was used in every protocol (except of course A). However the protocols differed in how those three 3D structures were mutually aligned. For protocols B, C, and F, individual template conformations were generated with standard Sybyl-X [19] modeling tools, minimizing the Concord structure with the MMFF force field. These three template conformations were superimposed by two means, for protocol B and C as, respectively, the highest-scoring and 100^th^ ranked hypotheses produced by Surflex-Sim [20, 21] with all defaults, and for protocol F simply by manually translating the displayed protocol B structures, so that all heavy atoms in different structures were separated by at least three Angstroms. For protocol D, these input template alignments were the structures as generated and “posed” by Concord [22] directly. Fig 2 shows the template alignments that were generated for the hERG target by the B, C, D, and F protocols.

For protocols E and G, the template CoMFA alignment step was omitted; instead the 3D-QSAR model for E was “derived” from only the Concord-generated and “posed” training set structures, while, for protocol G, the 3D structures of the Concord-only training sets of protocol E were further randomized, by setting all the adjustable torsional angles to random values, then posing the result by positioning of three randomly selected heavy atoms, successively at the origin, along the X axis, and in the X-Y plane, and finished by a centering at the origin.

For comparison of the binary active/inactive performance of template CoMFA with that of the usual fingerprint methods, datasets with a more realistic proportion of inactive structures than ChEMBL provides were needed. The only public source of presumably inactive structures is the ZINC compilation. For each target, training and prediction sets were constructed by combining a random sample from ZINC with a random sample for that target from ChEMBL, in a 10:1 proportion for total counts of 2200 structures. This 10:1/ZINC:ChEMBL/inactive:active proportion was applied also to each template set by combining 30 structures randomly chosen from ZINC with the protocol B triplet, the ZINC structures being individually posed with the protocol B Surflex-Sim hypothesis by the Surflex-Sim align operation and assigned “CHEMBL_VALs” of 2.5 (weaker than millimolar affinity). The resulting template, training, and prediction sets were processed as previously described.

To directly compare the performance of a “Tanimoto/fingerprint” methodology with that of template CoMFA, the Tanimoto-predicted affinity of each prediction-set structure was taken to be the CHEMBL_VAL of its most Tanimoto-similar training-set structure. The fingerprints were Unity-2D.

Here are a few methods details. The default CoMFA process generates a “region” (lattice of coordinates where field values are sampled) to enclose a parallelepiped 2 Angstrom beyond the x, y, and z extents of any training set structure. However, whenever necessary to overcome memory allocation failures (as these training sets are probably the largest to which CoMFA has ever been applied), in particular for the F and B^allTrng^ protocols, some regions were trimmed manually. It may also be noted that neutral protomers were used for all structures throughout this work, that tests of statistical significance were performed by Excel utilities, and that all other programs and scripts were extensions of SYBYL-X 2.1, written by the author, and for evaluation, completely and freely available via download, in its current alpha state packaged as SYBYL-X 2.2 [19].

## Results

Table 1 introduces the twelve biological targets. The X-ray template counts in the first column, the counts of downloaded structures, and the counts of structures actually used, have already been mentioned. A very rough indication of structural diversity, the counts of distinctive “reduced skeletons”, appears in the fourth column. Here, a “reduced skeleton” of a structure is obtained by removing all hydrogens, making identical the types of the other atoms and the bonds, and iteratively deleting terminal atoms until no more remain, leaving only generic ring systems and their connecting chains. (Thus structures lacking any ring have no reduced skeleton.) There are large differences among these reduced skeleton counts; apparently only a few ring systems have been explored for the GABAa target, while drug discovery challenges are surely the motivation for the over one thousand reduced skeleton varieties reported for hERG and facXa.

The standard deviations of these ChEMBL_VALUEs, appearing in the fifth column, are the starting base lines for this work, the results from the following null hypothesis: in the absence of additional information, the least uncertain prediction of the affinity for an unknown structure would be the average of known affinities, and the error of prediction would be the standard deviation of those affinities, the values shown in the fifth column. The criterion of success for any prediction methodology is the degree by which this uncertainty is reduced. As already mentioned, ChEMBL compilations lack inactive structures, so the SD of measured affinities among a truly representative selection of structures would be much larger.

With improved predictions of affinity being the goal for these studies, the key results are the bolded values in the next two columns, the uncertainties of template CoMFA model predictions using protocol B alignments of the input templates. The sixth column reports the standard deviations in the prediction errors of a random half of the CHEMBL_VALUEs, using a template CoMFA model trained on the other half. The seventh column provides an error estimate for an important application scenario, that of using a model trained on all known values to predict a single unknown, as the standard error of predictions during leave-one-out cross-validation. As discussed below, for all these models this estimate was found to be equivalent to the standard deviation of errors in truly prospective predictions.

Comparisons of these bolded values with those “null hypothesis” values in the fifth column indicate that the fundamental goal of these studies was achieved. For every target studied, the protocol B template CoMFA models indeed reduced the uncertainty of CHEMBL_VALUE predictions, compared to the null hypothesis. The average of these twenty-four uncertainty reductions in CHEMB_VALUEs (log affinity units), the differences of values in the fifth column from the values in the sixth or seventh columns, is .286 (+/-.112) (p > 99.9%, according to a one-sided T-test)

**Fig 3:**
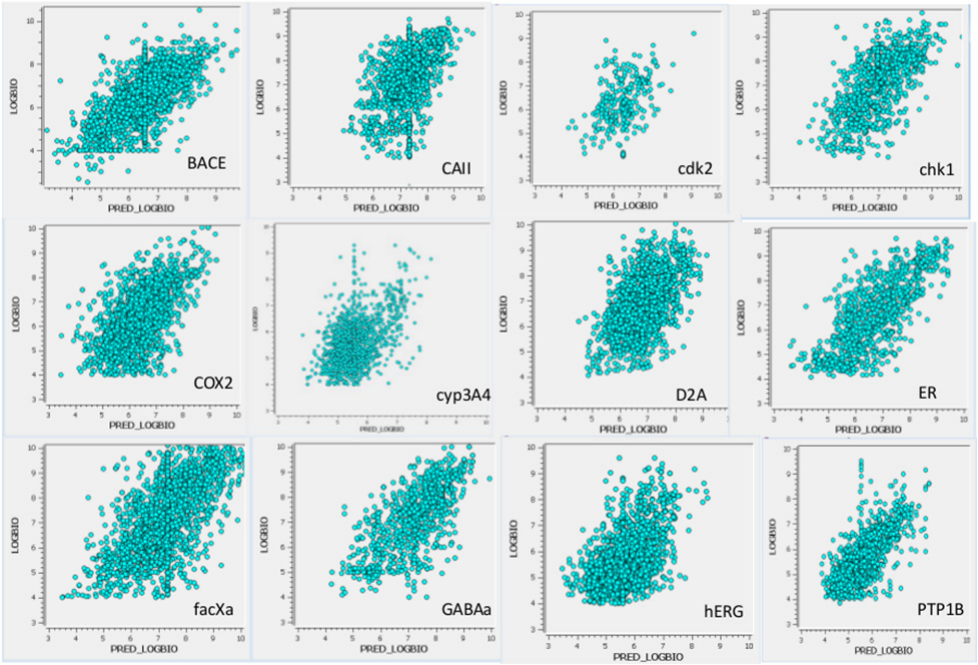
“Predicted vs. Actual” plots of the CHEMBL_VALUEs (log affinities) for the twelve targets.

Visual representations of the same results for each of the twelve targets are provided as “actual vs predicted” plots in Fig 3. Please note that, because most of their thousands of data points are buried in the centers of these plots, these images may convey an impression of a lower average degree of fit than actually exists.

The variability of the training data, the measured biological responses, puts two lower bounds on the SDEP values that should be achievable by these models. One is the standard deviation of biological affinities obtained from within-laboratory repeat measurements for the same compound, usually assumed to be 0.3. However, the ChEMBL compilations include many cross-laboratory measurements for the same compound, which, as an additional source of variability, increase the lower bounds of the potentially model-achievable SDEP. As a rough estimate of this lower bound, the CHEMBL_VALUEs in the above-mentioned tabulations of paired duplicate measurement were regressed against one another, and the resulting *s* values averaged, yielding 0.70 (+/- .25) as the best (least uncertain) SDEP that any CHEMBL_VALUE-derived predictive model should be capable of yielding.

Biological affinity is often found to correlate with molecular weight, a possibility which if ignored can misdirect lead optimization programs [23] and trivialize lengthy docking calculations [24]. Therefore, as a negative control, the eighth column of Table 1 reports the SDEP values from the correlation of the predicted CHEMBL_VALUEs with molecular weight. Comparison of these SDEPs with those in the fifth column does suggest slight molecular weight correlations for perhaps three of the twelve targets. However, comparisons with the sixth or seventh column indicate the 3D-QSAR, shape-specific, components of these template CoMFA correlations to be far more significant (p>99.9% according to a one-sided T-test).

A more meaningful comparison of template CoMFA is with the dominant methodology for off-target prediction, the degree of structural similarity as expressed by the Tanimoto coefficient of structural fragment occurrences [25, 26]. For this comparison, as described within Materials and Methods, each unknown CHEMBL_VALUE was “predicted” to be that of its most Tanimoto-similar structure (or “nearest neighbor”) having a known CHEMBL_VALUE. The SDEPs from applying this process to each of the twelve targets, appearing in the ninth column of Table 1, are hardly distinguishable from the template CoMFA SDEPs in column six or seven shows, with an average “superiority” of the template CoMFA SDEPs being a statistically meaningless .005 log units.

However, despite this resemblance in overall statistical quality, because the methodological foundations of the methods differ, the two methods often make different predictions for individual structures. An attempt to quantify this observation appears in the tenth column of Table 1. These values are the r^2^ values from the correlation of the SDEPs from template CoMFA with those from this expression of Tanimoto fingerprint coefficients. The average of these values is .227 (+/-.048), which can be compared with the average of .664 (+/- .110) for the r^2^ values from the twelve protocol B models, suggesting that differences between the affinity predictions of the two methods are substantive and numerous.

The rightmost column of Table 1 reports the degree of similarity between the best and the 100^th^ best Surflex-Sim alignments (protocols B and C), as the RMS of distances between corresponding heavy atoms, after a rigid-body fit of the two alignments. Though of course highly variable, and also upward biased, for example by ignoring alternative topological mappings from symmetry, the average difference of 4.6 Angstrom in these twelve RMS values surely indicates that statistical similarities between the models from the B and C protocols are not simply a matter of conformational similarity in their underlying alignments.

Table 2 provides supporting statistical metrics for one of the—template CoMFA models derived by applying the seven protocols to the CHEMBL_VALUEs for the facXa target.

**Table 2:** BACE (Beta-secretase 1): Template CoMFA model properties.

Input Data Protocol Code*	Model Derivation Metrics					SE of Model Predictions
	q^2^	SDEP	# cp	r^2^	s	
**A**	0.493	0.87	12	0.738	0.63	0.88
**B**	0.522	0.98	9	0.780	0.66	0.87
**B^allTrng^**	0.603	0.78	17	0.747	0.62	NA
**B^halfTrng^**	0.358	0.99	7	0.720	0.65	0.75
**C**	0.511	0.86	12	0.744	0.62	0.88
**D**	0.245	1.07	4	0.426	0.93	1.26
**E**	0.232	1.08	4	0.469	0.90	1.15
**F**	0.764	0.93	12	0.880	0.66	0.87
**G**	0.248	1.08	4	0.428	0.93	1.13

*A = Xray templates; B = three Surflex SIM best scoring templates (standard protocol); Balltrng = B trained on all structures; B = halfTrng = B trained on quarter of structures; C = as B for 100th best Surflex-SIM alignment; D = Concord templates; E = no templates (Concord only); F = as B, separated templates; G = as E, Concord then randomized.

Some of the tabulated metrics may be unfamiliar. As is almost universal for 3D-QSAR with its extremely numerous field-based descriptors, template CoMFA models are derived by partial least squares (PLS) [27]. The effectiveness of PLS in generating robust linear models from many times more descriptor columns than data point rows, an impossible task for multiple linear squares regression (MLS), results from its treatment of those descriptors as an internally invariant block rather than a collection of independently manipulated columns. This descriptor block is iteratively rotated, scaled, and pruned to maximize its cumulative coincidence with the dependent variables (here the CHEMBL_VALUEs). The result of each iteration is accumulated into the PLS model, whose improvement after each iteration is assessed by cross-validation, usually leave-one-out, generating an SDEP and a “cross-validated r^2^” (conventionally abbreviated as q^2^). Each iteration thereby adds another “component” to the PLS model; iteration ends when the SDEP stops declining and/or the q^2^ value stops increasing. The final PLS model is functionally equivalent to a best-fit MLS model, accompanied by r^2^ and s metrics and, in analogy to the number of MLS coefficients, the final number of PLS components. The final q^2^ and SDEP values are also recorded, as indicators of the PLS model’s likely predictive performance. The first five columns in Table 2 provides these q^2^, SDEP, component counts (labelled as “#cp”), r^2^, and s values from application of each alignment protocol to the facXa target.

The sixth and rightmost column in Table 2 reports the prospective predictive performances of the facXa template CoMFA models, as the SE of the differences between the predicted and actual CHEMBL_VALUEs. It is noteworthy that, contrary to the usual QSAR experience [28], the SDEPs from cross-validation (second column in the table) are excellent predictors of these SEs from actual predictions, with the average value for the 108 models being -0.004 (+/-0.089). However the unusual size and structural diversity of these training and prediction sets helps to account for the agreement between them. This agreement provides, firstly, very strong evidence for a remarkable soundness of these models, and of their behaviors, some highly counter-intuitive, and, secondly, a further justification, again only for these presumably uniquely large and diverse data sets, of the proposition that the cross-validation SDEP is a robust estimator of the errors for that important application scenario, prediction of a single unknown using a model trained on all known values (seventh column of Table 1).

**Fig 4:**
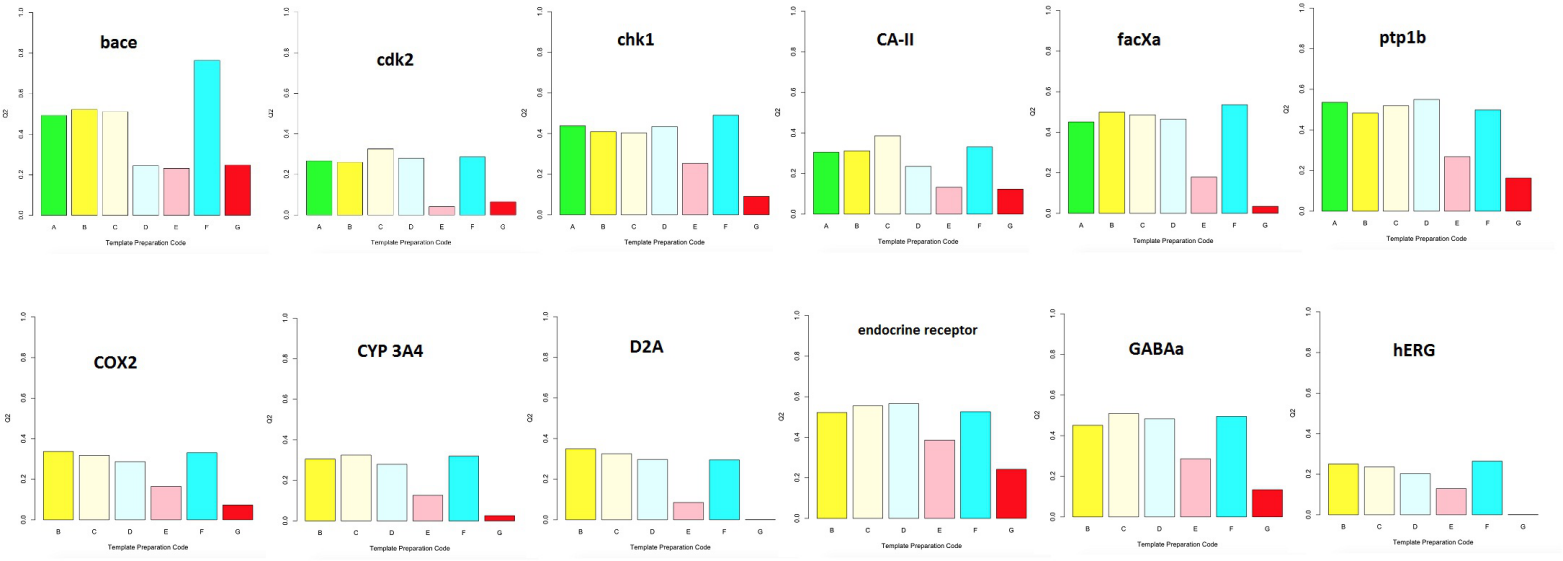
Bar chart representation of the q^2^ values for the models from all combinations of template protocols and targets. Yellow bars are from Xray templates (protocol A), green from Surflex-SIM alignments (B and C), cyan from the “irrational” Concord (D) or spatially dispersed (F)alignments, and red from omissions of template CoMFA (E and G).

Before presenting some comparisons among the model qualities of the seven different template protocols, it must be emphasized that these comparisons are similar, again remarkably so, across all twelve targets. To help convey this crucial finding, Fig 4 provides bar charts that represent the q^2^ values for the models from all combinations of protocols and targets. Details of all these models, presented as for the facXa model in Table 2, may be found within the Supporting Information.

Most molecular modelers would use either protocol A or B to generate the aligned 3D template structures required to perform template CoMFA. Protocol A, the direct use of the crystallographic coordinates from every target-bound ligand, would probably be the first choice. However, when such coordinates are not available, here for half of the targets, protocol B becomes the most likely alternative. In protocol B, the template alignments are constructed by seeking a mutually maximal “shape similarity”, applying one of several programs that varyingly include such specific “shape” aspects as superposition of key atoms, external ligand field intensities and/or extrema, and collective occupancy volume. Different programs will evidently generate different template alignments, which for template CoMFA application will produce different models and different predictions. Which alignments and models should be preferred, and why?

This concern, the effect on template CoMFA of this unavoidable uncertainty among the underlying template alignments, was a starting point for these studies. Target-bound ligand structures were available for six targets. The alignment of target-bound ligands, protocol A, might or might not somehow be “optimal”, but certainly represents a fixed standard for comparisons. Starting with the same set of possible templates, how different from their protocol A-based template CoMFA model would protocol B-based models be? The program available to the author for combinatorially seeking optimal alignments was Surflex-SIM [20, 21], whose elegant representations of shape similarity do involve lengthy refinement. Therefore in these studies, the template sets for all protocols except A were limited to three ligands. This did seem far too little 3D information to successfully align such a large variety of training set structures. But, completely unexpectedly, not only were three templates sufficient, but also, for every target, the results reported here are all from the first trio of templates tried.

The first two lines of Table 2 introduce another counter-intuitive result from these studies. For every one of these six targets, there is little difference between the statistical properties of the protocol A and protocol B template CoMFA models. There is negligible support for the expectation that crystallographically-based alignments should usually be more effective than these shape similarity-based alignments. While the first A-B column of Table 3, which provides summarized comparisons of q^2^ across all targets, does report an average q^2^ superiority of .001 for the crystallographic template protocol A, such a small difference is neither statistically nor practically significant. (As a further precaution, progressive Y scrambling [29] was applied to the HERG target, obtaining a smooth and almost horizontal plot of the q^2^/SDEP ratio vs number of components, confirming the highly robust character to be expected for any model derived from such a large and well-conditioned training set.)

**Table 3:** Comparisons of q^2^ values (from leave-one-out crossvalidation) for template CoMFA models, as produced by applying protocols A-G to the input data, averaged over the twelve biological targets.

	A	B	C	D	E	F	G	
**Average of q^2^**	0.346	0.392	0.408	0.360	0.190	0.406	0.098	
**Standard dev of q^2^**	0.207	0.102	0.104	0.130	0.098	0.108	0.087	
**Protocol Comparison**	A-B	A-F	B-C	B-D	B-E	B-F	D-E	E-G
**Average q^2^ Difference**	0.001	-0.025	-0.017	0.031	0.201	-0.014	0.164	0.094
**Standard dev of q^2^ diffs**	0.037	0.042	0.036	0.090	0.062	0.034	0.089	0.068
**Z.test of q^2^ difference**	0.478	0.926	0.947	0.113	0.000	0.924	0.000	0.000

**Fig 5:**
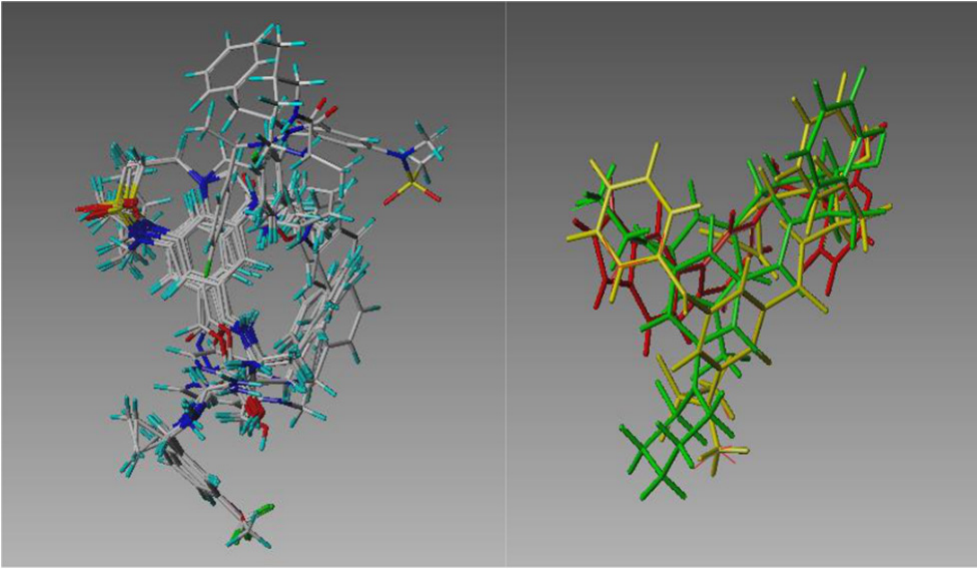
Comparison of input template alignments for the BACE target. The left panel depicts 13 ligands extracted from crystallographic structures of BACE. The right panel shows three of those ligands as optimally realigned by the Surflex-SIM program.

Fig 5 further provides, for the BACE target, a visual confirmation of the large differences between the crystallographically determined and protocol B-generated template alignments.

This finding raised the hopeful thought that the concern which had motivated these studies, the uncertainty of predictions starting from template alignments derived from shape similarity, might not be such a major challenge. So protocol C, Surflex-SIM’s 100^th^ best superimpositions of the same three ligands, was applied. Despite the substantial RMS differences between the best and 100^th^ best alignments, reported as previously mentioned in the rightmost column of Table 1, the corresponding template CoMFA models actually favor slightly the 100^th^ best alignment, by an amount just short of statistical significance (column B-C in Table 3). This lack of sensitivity by template CoMFA to the unavoidable uncertainty in the alignments of its input templates, though highly counter-intuitive, would be of great practical benefit. What are its limits?

To try to distinguish the relative roles of template CoMFA and Surflex-SIM in producing this unexpected consistency in model statistics, several additional protocols were tried. First, with protocol D, the effect of Surflex-SIM in aligning the three templates was removed, by simply using as the template alignment the shapes and poses that Concord produced from their 2D structures. The resulting B-D comparison in Table 3 shows that the Surflex-SIM alignments of the templates of their input Concord “alignments” do slightly improve the models, by an average q^2^ of .031 (+/-.090). Of course the training set structures, the ones from which these CoMFA models are actually derived, are still aligned by template CoMFA to these Concord-generated templates. So next, in protocol E, template CoMFA alignment was also omitted, by deriving each CoMFA model from a training set whose structures were “aligned” only in ther initial Concord poses. The resulting B-E decline in q^2^ of .201 (+/-.062; p >99.9%) confirms the decisive importance of template CoMFA alignments in yielding these robust models. Finally, two extremely unrealistic alignment protocols were tried, as detailed in Materials and Methods. For protocol F, the displayed templates from protocol B were manually separated completely from one another, while, for protocol G, every training set structure from protocol D was deformed in random fashion, both conformationally and positionally, and CoMFA was directly applied to these structures. As reported in Table 3, the F protocol separation of the templates actually produced the best average q^2^ of any protocol, and by a statistically significant amount (p>98.7%) if the A-F and B-F comparisons are pooled, while as expected the G randomization protocol produced the lowest average q^2^. Nevertheless the G protocol’s average q^2^ value is significantly greater than 0.0 (p>99.9%), probably because of underlying correlations between affinity and such other, non-shape-specific, descriptors as molecular weight.

**Fig 6:**
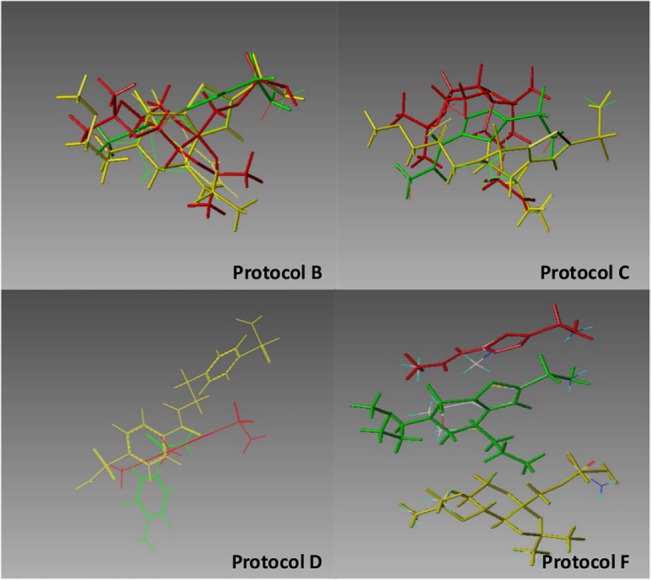
Input template alignment alternatives for the carbonic anhydrase II target. The three structures in each image are the ligands extracted from .pdb files 1BN3 (green), iBNM (yellow), and 1BNN (red). Each one-letter label refers to the protocol that generated the alignment. The protocol F image also includes the best-matching 3D structure, from within the training set, that the template CoMFA program generated for each of these three templates.

A visual impression of the templates that the B, C, D, and F protocols produce for carbonic anhydrase II appears in Fig 6.

A second key dimension of input for a template CoMFA model, the number of structures in the training set, was also explored. Neither varying the means of randomly dividing the ChEMBL_VALUEs into equally sized training and prediction sets, nor exchanging the roles of training and prediction sets, had any effect on the statistics of the template CoMFA models (data not shown). But, as shown for example in the italicized B^halfTrng^ and B^allTrng^ lines of Table 2, halving the size of the training set (for the 50% larger prediction set) weakened a model’s performance by 10%, according to a comparison of the average SDEP increase of .093 log units (+/-.121; p>99.5% according to a Z test) with the average SDEP of 0.89 for these models. Conversely, training these models on all rather than half the CHEMBL_VALUEs improved their performances by 10%, compared to the average SDEP decrease of .089 log units (+/-.041; p>99.9% according to a Z test).

Comparison of these template CoMFA results with those that those produced by the familiar Tanimoto 2D fingerprint methodology is the subject of Table 4.

**Table 4:** Comparison of Correct Prediction Rates, between Template CoMFA and Nearest Neighbor Tanimoto 2D Fingerprint.

	ChBL >3.5 <4.5	Template CoMFA (TC)				NN, Tanimoto Fgpt				Diff, TRUE Pred, (TC—NN)	
		Inactive		Active		Inactive		Active			
Target		n	TRUE	n	TRUE	n	TRUE	n	TRUE	F Neg	F Pos
**bace**	10	1923	0.982	144	0.951	1992	0.997	201	0.950	-0.015	0.001
**cdk2**	8	1900	0.973	101	0.861	1983	0.991	199	0.839	-0.018	0.022
**chk1**	9	1845	0.965	131	0.763	1993	0.988	196	0.842	-0.023	-0.078
**CAII**	6	1825	0.965	104	0.702	1973	0.990	219	0.781	-0.025	-0.079
**COX2**	9	1920	0.969	109	0.862	1988	0.991	200	0.850	-0.022	0.012
**cyp3A4**	5	2041	0.954	35	0.971	2000	0.976	177	0.729	-0.022	0.243
**D2A**	7	1861	0.960	105	0.771	1977	0.984	223	0.744	-0.025	0.027
**ER**	12	1880	0.969	102	0.873	1990	0.993	204	0.775	-0.025	0.098
**facXa**	12	1838	0.978	143	0.769	1993	0.988	196	0.842	-0.011	-0.073
**GABAa**	11	1700	0.971	165	0.606	1988	0.992	209	0.880	-0.021	-0.274
**hERG**	7	2028	0.949	34	0.912	2026	0.971	166	0.777	-0.022	0.135
**ptp1b**	10	2030	0.962	60	0.967	1989	0.990	174	0.839	-0.029	0.128
**Average**		1899	0.966	103	0.834	1991	0.988	197	0.821	-0.021	0.013

For this purpose, as described above, to better represent the preponderance of inactive structures in typical applications, the training and prediction sets became 10:1 inactive:active proportions, with the inactive 2000 members of each randomly drawn from ZINC and assigned “CHEMBL_VAL”s of 2.5 and the active 200 members random drawings from the ChEMBL compilations for that target. Also, the continuous binding affinity values that are predicted by template CoMFA were converted into a three-way active/inactive/uncertain classification scheme by assigning “activity” to all structures having a CHEMBL_VAL greater than 4.5, “inactivity” to those with a CHEMBL_VAL lower than 3.5, and “uncertainty” to the remaining structures. The counts of those “uncertain” structures are given for each target in the first column of Table 4, from among the total of 200 from ChEMBL.

The results important for methodology comparison are in the two blocks of four columns (second through the ninth) in Table 4. Each block reports the critical elements from the contingency table for active/inactive predictions, the left block for template CoMFA and the right block for the Tanimoto method. Within each block, the four columns report, the first pair for “inactive” and the second for “active” classified structures, the count of predictions attempted and the fraction of those predictions that were correct. (The discrepancies between the perhaps expected prediction counts of 2000 and 200 and those actually reported have several causes. No prediction was made for structures whose ChEMBL_VAL is between 3.5 and 4.5, and ChEMBL_VALs less than 3.5 of course classify a structure as “inactive”, to be included with the 2000 ZINC structures. And predictions sometimes failed, particularly from template CoMFA when Concord did not produce the valence geometry needed as a starting point.) The final comparisons occupy the rightmost pair of columns. The first comparison shows that for “inactive” structures, for every target, the Tanimoto classification of the ~2000 structures is better than that from template CoMFA, with an average superiority of .0214 (+/-.0048; p >>99.9%). The second comparison, for the many fewer “active” structures, reports an inconclusive comparison, the overall trend perhaps instead slightly favoring the template CoMFA classification, but by an average of .0134 (+/- .1331), not of any remotely statistical or practical significance.

However, it is surely of more practical interest to investigate how productively these two methodologies might combine, rather than compete. The results of such studies are presented in Table 5.

**Table 5:** Results from OR'ing or AND'ing the predictions of Template CoMFA with Tanimoto NN predictions.

	OR'd hit increase		AND'd Correct Prediction Increase					
			Inactive		Active		TC&NN—NN	
Target	n+	n>%	n	TRUE	n	TRUE	F Neg	F Pos
**bace**	3	30	1877	0.998	134	1.000	0.000	0.050
**cdk2**	4	13	1824	0.995	85	0.929	0.004	0.090
**chk1**	9	29	1770	0.989	93	0.978	0.000	0.137
**CAII**	4	8	1732	0.992	72	0.958	0.002	0.178
**COX2**	1	3	1845	0.991	94	0.989	0.000	0.139
**cyp3A4**	3	6	1921	0.978	31	1.000	0.002	0.271
**D2A**	10	18	1759	0.990	74	0.959	0.006	0.215
**ER**	4	15	1802	0.986	84	0.988	-0.008	0.214
**facXa**	7	23	1772	0.999	104	0.990	0.010	0.149
**GABAa**	1	4	1646	0.993	103	0.951	0.000	0.071
**hERG**	24	65	1926	0.993	45	1.000	0.022	0.223
**ptp1b**	6	21	1950	0.997	72	0.986	0.006	0.147
**Average**	6	20	1819	0.992	83	0.978	0.004	0.157

**Fig 7:**
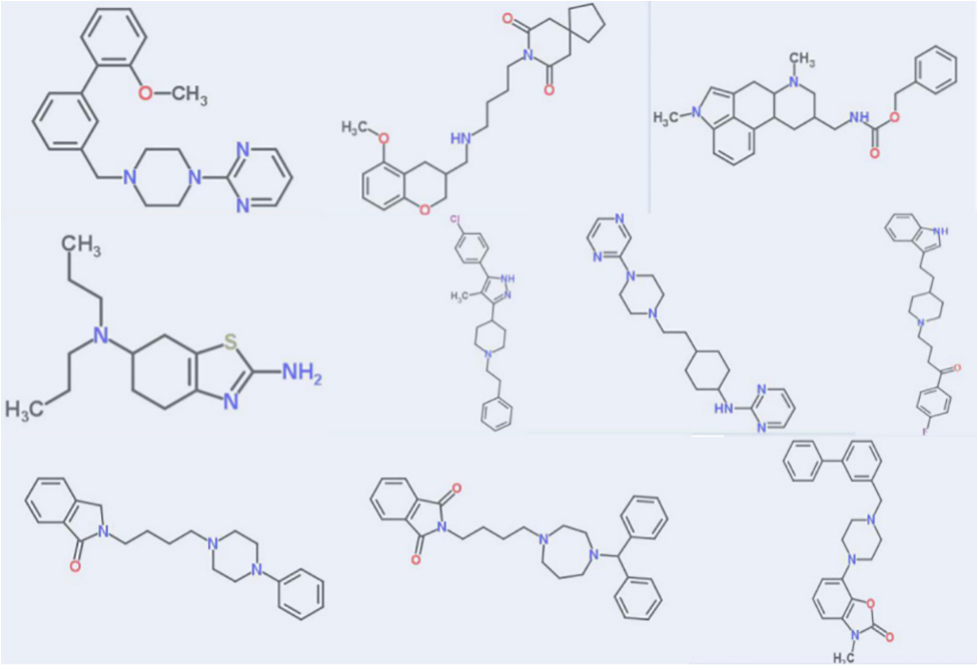
Structures with high binding affinity to the D2A target. These identified as D2A “actives” by the template CoMFA method (3) reported as D2A “inactives” by the Tanimoto 2D fingerprint nearest neighbor method.

One of two obvious possibilities is that template CoMFA, as a fundamentally different methodology, will identify “active” structures that the Tanimoto methods overlook. The first two columns of Table 5 confirm this possibility. The first of these is the count of such additional “true active” structures among these ~200 predictions, as provided by template CoMFA, for each of these targets. The second converts this count into an incremental percentage of the count of true actives identified by the Tanimoto method. The average incremental percentage is 15%. To provide an impression of the structural diversity of such structures, the ten additional D2A binders re depicted in Fig 7.

The other possibility is that the error rates in distinguishing active from inactive structures will be lower for predictions that template CoMFA and a Tanimoto method agree on, expected again because of the fundamental independence of these methodologies. So for only those (though of course still numerous) active structures, the previous tabulation of counts and success rates for inactive and active predictions is repeated in the next four columns of Table 5. The last two columns of Table 5 provide the critical results, the drop in these error rates when template CoMFA predictions agree with the Tanimoto predictions, for false negatives and false positives respectively. More exactly, these values are the differences between the values in the bolded columns in Tables 4 and 5. Evidently the error rates whenever template CoMFA predictions confirm those from Tanimoto NN are consistently lower, the average improvements being .0038 (+/-.0072; p>95.0% according to a Z-test) for the “inactive” class and .1569 (+/- .0665; p>>99.9%) for the “active” class.

## Discussion

The introductory questions that these studies addressed provide the framework for discussing the findings.

### Can template CoMFA models be obtained from training sets whose structures mostly lack any obvious homologies whatsoever?

Twelve satisfactory models, each including all the appropriate data available from ChEMBL for training or prediction, were obtained for every one of twelve biologically diverse targets. If only because of the dependence of these models’ existence on template CoMFA’s automatic alignments, these are likely much the largest and most structurally heterogeneous 3D-QSAR models ever published. For any experienced practitioner of existing 3D-QSAR methodologies, starting with the author, this key finding strains credibility.

A caveat needs repetition. The structural scope of any method for predicting biological effects is limited by the diversity and balance of their training sets. The ChEMBL compilations are strongly biased to include active rather than inactive structures. Thus these particular models may be less effective in distinguishing a few active structures among a large population of inactives. Nevertheless, wherever high-throughput screening has provided more balanced training sets, these results would seem strong justification for trying template CoMFA on them.

### Can stable and robust template CoMFA models be obtained from input templates whose alignment is purely ligand-based, and therefore inherently more uncertain and subjective, than X-ray-aligned input templates?

Simple inspection of a static target-bound ligand structure, while not overlooking the thermally-necessitated dynamic character of the actual biological environment and the selectively modulated functional behavior required for that target [30], suggests the huge challenges that any prediction of biological activity seeks to overcome. Crystallographic structures do at least provide, as assurance, well-defined templates as starting points. The only alternative, aligning ligands in isolation by maximizing their overall physicochemical similarity, by a truly objective and unbiased means such as Surflex-SIM in these studies, usually generates hundreds of reasonable starting points. Nevertheless, simply using, as the only “3D input”, the templates, three arbitrarily selected ligands in their highest scoring Surflex-SIM alignment, produced these statistically satisfactory template CoMFA models in these twelve of twelve trials. Moreover, for the six targets having X-ray structures, the differences in statistical quality between X-ray and ligand-only template CoMFA models were completely negligible. Such unexpected findings are also encouraging evidence for a wide scope of applicability for template CoMFA.

### How dependent are ligand-derived template CoMFA models and their predictions on the number or variety of those input templates?

The contents of Tables 2 and 3 show this dependency to be very low and again astonishingly so, although some rather small differences between protocols do achieve statistical significance because of their remarkable consistency. Models based on the100^th^ best Surflex-SIM template hypotheses actually are a bit better than the best hypotheses (protocol B vs. protocol C), though to a small and not quite statistically significant degree (B-C in Table 3). Even using as templates the Concord structures, in whatever superposition happened to be produced (protocol D), did not weaken the models to a statistically significant degree. Of course, it is the template CoMFA alignments of the training sets to the templates that enabled and distinguished these studies, and so as expected the model statistics deteriorate significantly when this procedure is omitted (protocol E), and even more so when the Concord-generated structures from protocol E are intentionally randomized (protocol G). Finally, completely separating the templates from one another provided the best model statistics (protocol F).

So the best model statistics result from the templates-separated protocol, one that most conspicuously lacks any obvious physicochemical justification–yet another finding that is troublingly counter-intuitive, and sometimes contrary to much of our collective decades of QSAR experience. Yet the remarkable number, consistency, size, structural scope, and, in particular, the predictive performances of these template CoMFA models surely demand serious attention. And there does exist a single rationale which all of these current results support. However this unorthodox viewpoint, whose slow emergence paralleled the author’s development of alignment methodologies, has been difficult to adequately explain [31, 32] [13]. So here is another attempt.

This viewpoint can be summarized as: “*For the purposes of generating predictive 3D-QSAR models*, *alignments that limit underlying field variations to those directly caused by explicit structural variations*, *and* *which therefore maximize the ratio of signal to noise within those field variations*, *are at least as effective as alignments which are guided only by physicochemical realism*”. As an example, consider the 3D-QSAR modeling of the affinities to an X-ray established target by a combinatorial library, composed of structures that are identical except for a particular side chain. In this situation, many modelers, desiring to maximize physiochemical realism, would dock each structure in the library into the known target structure to generate its individual alignment. During docking, all atoms, including those which are structurally invariant, will move, to locations which will differ as the side chains are exchanged. Even small variations in the location of any atom can have immense effects on field intensities at its nearby lattice points, particularly steric, which of course then affect the resulting 3D-QSAR model. And very likely most of these variations in the locations of invariant atoms have no systematic dependence on the side chain variations that are the actual cause of the affinity variations that the model is intended to explain. Any such non-systematic dependencies produce noise within the field variables, opposing any signal, from which a 3D-QSAR model is to be derived. Noise in the input data is particularly detrimental to model-building when partial least squares is the model generator [32].

So this unorthodox viewpoint instead prescribes, “Wherever, within a training or prediction set, the structures are identical (or very similar), do not allow the alignment protocol to alter the locations of individual atoms.” This restriction limits the variation among the field variables to those lattice points that are spatially adjacent to atoms whose synthetic alterations must be the only actual causes of the changes in biological response that a 3D-QSAR model is intended to explain.

But, how can this viewpoint explain any of the current results, which involve training and prediction sets that are as different in their composition from combinatorial libraries as can be imagined? An answer can begin with two observations. First, the particular candidate-to-template atom chain match that template CoMFA chooses while generating a candidate alignment is the one which maximizes the count of matching atoms, those whose coordinates template CoMFA then simply copies and hence are certainly invariant. Second, the topomer canonicalization, which positions the atoms in unmatched candidate side chains, was developed with the goal of placing similar side chain atoms in similar positions relative to the side chain root. Thus the critical role of template CoMFA alignment in the protocols that yield the better CoMFA models is understandable. However, the effectiveness of template CoMFA in those roles is a pleasant surprise. With only three templates that each of these diverse candidates can be matched to, many of the atom chain matches and resulting candidate alignments that template CoMFA produces do seem preposterous to physicochemical intuition. Yet, this consistency in obtaining models from these template CoMFA alignments, irrespective of targets, template alignment protocols, and training set compositions, rather conclusively establishes the relevance of these candidate alignments, at least on average.

The irrelevance of the template inputs themselves, that is, the so far universal ability to obtain a satisfactory model for a given target with a few arbitrarily chosen and arbitrarily aligned 3D structures as templates, is another big surprise. From a practical perspective, by removing any requirement for some complicated and uncertain template selection methodology, this surprise is a welcome one. However, with template selection thus becoming a degree of freedom, the credibility and interpretability of a model will surely benefit if target-bound ligand structures, experimental or docked, are used as the template inputs for template CoMFA whenever a target structure is available.

Another benefit of this template-independence may be in further stabilizing the robust platform that template CoMFA’s objectivity already offers for further 3D-QSAR method development (such as the current work represents). There are many parameters hidden within template CoMFA, currently having fixed and arbitrary (though fortunately productive) values, whose systematic exploration should be made much more productive by this template-independence.

However, the dependence of the affinity prediction for an individual structure on the template alignments used to build the model, as contrasted with this stability of overall model statistics, still requires investigation.

### How dependent are the models and their predictions on the size of their training sets?

These findings are straightforward as well as consistent, as reported for example in the B-B^allTrng^ and B-B^halfTrng^ rows in Table 2. Doubling the size of the training sets reduced their uncertainty of affinity prediction by an average of .08 (+/.05) log units; halving their size increased their uncertainty of affinity prediction by an average of .10 (+-.11) log units.

### How accurate are template CoMFA predictions?

For the all-ChEMBL models (B^allTrng^), the LOO q^2^ averaged over these twelve targets is .476 (+/-.096) and the associated SDEP is 0.89(+/-.12). Although the accuracy of any particular prediction obviously depends on the target, the training set, and the frequency of discontinuous or so-called “magic methyl” behavior, this objective means to reduce the uncertainty of an affinity prediction by almost 50% (i.e., the .476 LOO q^2^) compared to “the average of all affinities so far measured” would seem helpful in many drug discovery contexts.

Can the uncertainties of template CoMFA predictions be further reduced? Until now, the overwhelming barrier to 3D-QSAR improvements, the unavoidable subjectivity of training set alignment hypotheses, had discouraged the author from pursuing ideas that could only yield relatively marginal improvements. In the context of template CoMFA, these possibilities include manipulation of auxiliary similarity indices, consensus scoring while varying the template hypothesis, and modifying template alignments to minimize conflicting field effects at critical lattice points (probably the cause of protocol F’s superior model statistics).

However, access to more commensurate training data than can be provided by ChEMBL and other such compendia may be necessary. As mission-critical to the performance of these studies as the ChEMBL data base has been, the heterogeneities of its sources put a relatively high floor on how far its training sets can reduce the uncertainties of prediction. The above-suggested lower bound estimate of that floor, the variance in the affinities reported by ChEMBL for the same structure, is 0.70 (+/-.25), not so far below the current SDEP for affinities of 0.89 using template CoMFA. An indication that this inherent floor actually might approach the 0.89 value even more closely is a very strong correlation (r^2^ = 0.84) between the SDEP’s resulting from the two methodologically independent approaches, template CoMFA and Tanimoto coefficients (seventh and ninth columns of Table 1).

### How accurate are template CoMFA predictions? How do its predictions compare with those from the established approaches based on substructure frequencies and counts?

First, it should again be cautioned that, for all ChEMBL’s unique and critical virtues, the biases of its content make it poorly suitable for the primary application of substructural approaches, distinguishing a few structures active at some target of interest from a much larger number of inactive ones. More appropriate data sets would comprise measured affinities for a large number of structures, such as “high-throughput screening” can yield, but few if any of these are publicly available. Therefore to address these questions, data sets having more relevant SAR distributions had to be somewhat artificially constructed, in a way that may somehow distort the results. For example, the prediction success rates in Table 4 seem much better than those reported, for example, in publications of success in virtual screening.

Nevertheless, taken at face value, the results in Table 4 are straightforward. In particular, when compared as competing stand-alone methodologies, for these data sets the Tanimoto method is indisputably the more selective, reducing the frequency of false positives (i.e., inactives misreported as actives) to half that frequency from template CoMFA. However, in sensitivity (avoiding false negatives, or actives reported as inactives) the performances of the two methods cannot be distinguished; a slight superiority of template CoMFA is neither statistically nor practically significant. This similarity in sensitivities is consistent also with the ninth column in Table 1, reporting very similar accuracies of affinity prediction from template CoMFA and Tanimoto/fingerprints for the “active” ChEMBL structures.

However, as a practical matter, relative performance has little relevance to the potential value of template CoMFA for drug discovery. What seems of more interest is whether inclusion of this emerging template CoMFA can improve on the established Tanimoto approaches. The results in Table 5 suggest some benefit. If a project’s goal is to identify additional structures having affinity to some desirable target affinity, particularly if the target structure is unknown, or to better anticipate the profile of some structure of interest against a panel of biological targets, the third column of Table 5 reports that template CoMFA can identify substantially more true positives than does this Tanimoto method alone, and the specific examples of such retrievals in Fig 7 indicate the potential importance of such additional retrievals. Or, considering the expense and delay of any perhaps unnecessary assay, the reduction by a third in the frequency false positives (from 1.2% to 0.8% according to column seven in Table 4 and column four in Table 5, respectively) by combining template CoMFA with the template method seems useful.

**Fig 8:**
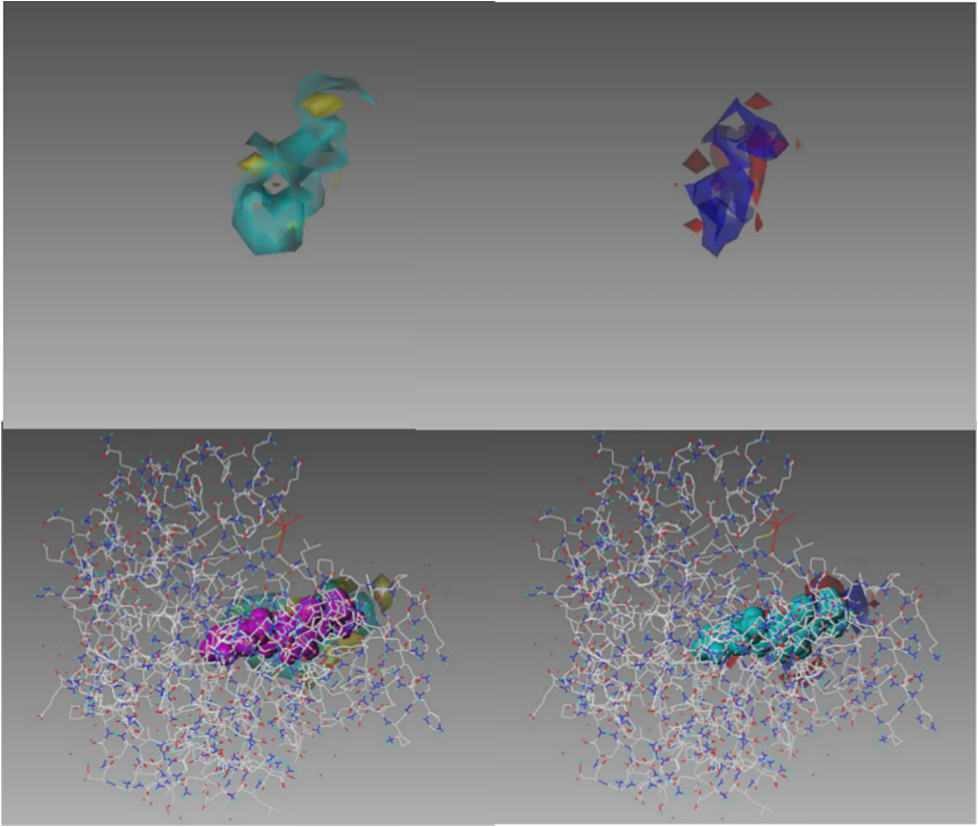
Additional representations of the protocol B template CoMFA model for carbonic anhydrase II. The upper panel shows the conventional “stdev*coeff” representation of a CoMFA model: the green shapes enclose spatial regions where affinity increases with steric bulk; the yellow shapes enclose regions where decreased steric bulk improves affinity; the red shapes enclose regions where more positive electrostatic potential improves affinity; the blue shapes enclose regions where more negative electrostatic potential improves affinity. The lower panel also includes structures of carbonic anhydrase II (skeleton representation) and the template CoMFA alignments of three highly active training set ligands (space-filling representations).

Template CoMFA also seems to offer less quantifiable benefits. One is that affinity is the endpoint for template CoMFA but at best only inferable from a fingerprint-based similarity approach. And degree of affinity is not only the actual and immediate determinant of any on- or off-target physiological response, it can also merge with pharmacodynamic modeling to provide dosage and other therapeutic guidances, in a way that structural similarity alone cannot. Another benefit is the much greater interpretability of a template CoMFA model. As suggested by Fig 8, the mechanistic and shape-specific nature of template CoMFA models, particularly in combination with other information such as target structure, can provide direction to the search for improved medicines.

In summary, here are some concluding thoughts.

The unusual breadth and depth of these studies and the remarkable consistency of their findings seem strong evidence that inclusion of template CoMFA can improve capabilities for off-target side effect prediction.

Although not explicit in this account, template CoMFA also has ease of use and versatility (having been developed with different applications in mind) that are superior to those of most other computer-aided molecular design methodologies.

The many findings from this work that were unexpected, at least to the author, suggest that some of the intuitive assumptions that guide the usual practices of computer-aided molecular design might be critically reconsidered.

## Supporting Information

S1 File Statistical parameters for template CoMFA models from all combinations of template protocols and targets. The text file provides tables analogous to Table 2 for all twelve targets, in csv format suitable for Excel.
(CSV)

S1 Text Structures of BACE ligands. The zip file contains, in. sdf format, the aligned structures, from the A, B, C, D, and F protocols, of all templates and training sets for the BACE target. The content of each file is evident from its name.
(ZIP)

S2 Text Structures of CAII ligands. The zip file contains, in. sdf format, the aligned structures, from the A, B, C, D, and F protocols, of all templates and training sets for the carbonic anhydrase II target. The content of each file is evident from its name.
(ZIP)

S3 Text Structures of cdk2 ligands. The zip file contains, in. sdf format, the aligned structures, from the A, B, C, D, and F protocols, of all templates and training sets for the cdk2 target. The content of each file is evident from its name.
(ZIP)

S4 Text Structures of chk1 ligands. The zip file contains, in. sdf format, the aligned structures, from the A, B, C, D, and F protocols, of all templates and training sets for the chk1 target. The content of each file is evident from its name.
(ZIP)

S5 Text Structures of COX2 ligands. The zip file contains, in. sdf format, the aligned structures, from the A, B, C, D, and F protocols, of all templates and training sets for the COX2 target. The content of each file is evident from its name.
(ZIP)

S6 Text Structures of CYP 3A4 ligands. The zip file contains, in. sdf format, the aligned structures, from the A, B, C, D, and F protocols, of all templates and training sets for the cyp 3A4 target. The content of each file is evident from its name.
(ZIP)

S7 Text Structures of D2A ligands. The zip file contains, in. sdf format, the aligned structures, from the A, B, C, D, and F protocols, of all templates and training sets for the D2A target. The content of each file is evident from its name.
(ZIP)

S8 Text Structures of ER ligands. The zip file contains, in. sdf format, the aligned structures, from the A, B, C, D, and F protocols, of all templates and training sets for the endocrine receptor target. The content of each file is evident from its name.
(ZIP)

S9 Text Structures of facXa ligands. The zip file contains, in. sdf format, the aligned structures, from the A, B, C, D, and F protocols, of all templates and training sets for the factor Xa target. The content of each file is evident from its name.
(ZIP)

S10 Text Structures of GABAa ligands. The zip file contains, in. sdf format, the aligned structures, from the A, B, C, D, and F protocols, of all templates and training sets for the GABAa target. The content of each file is evident from its name.
(ZIP)

S11 Text Structures of hERG ligands. The zip file contains, in. sdf format, the aligned structures, from the A, B, C, D, and F protocols, of all templates and training sets for the hERG target. The content of each file is evident from its name.
(ZIP)

S12 Text Structures of ptp1b ligands. The zip file contains, in. sdf format, the aligned structures, from the A, B, C, D, and F protocols, of all templates and training sets for the ptp1b target. The content of each file is evident from its name.
(ZIP)

S13 Text All input structures used for comparisons with Tanimoto. The zip file contains, for each target, the template, training set, and prediction set, as “2D” structures, in. sdf format.
(ZIP)
